# Supplementary material for: Genome-Wide Identification, Characterization, and Expression Analysis of the Copper-Containing Amine Oxidase Gene Family in Mangrove Kandelia obovata
Source: Int J Mol Sci. 2023 Dec 9;24(24):17312. doi: 10.3390/ijms242417312 (PMC10743698; doi:10.3390/ijms242417312)
Supplement: Supplementary file 1 [file ijms-24-17312-s001.zip › ijms-2738093-supplementary.pdf]

# Supplementary Materials

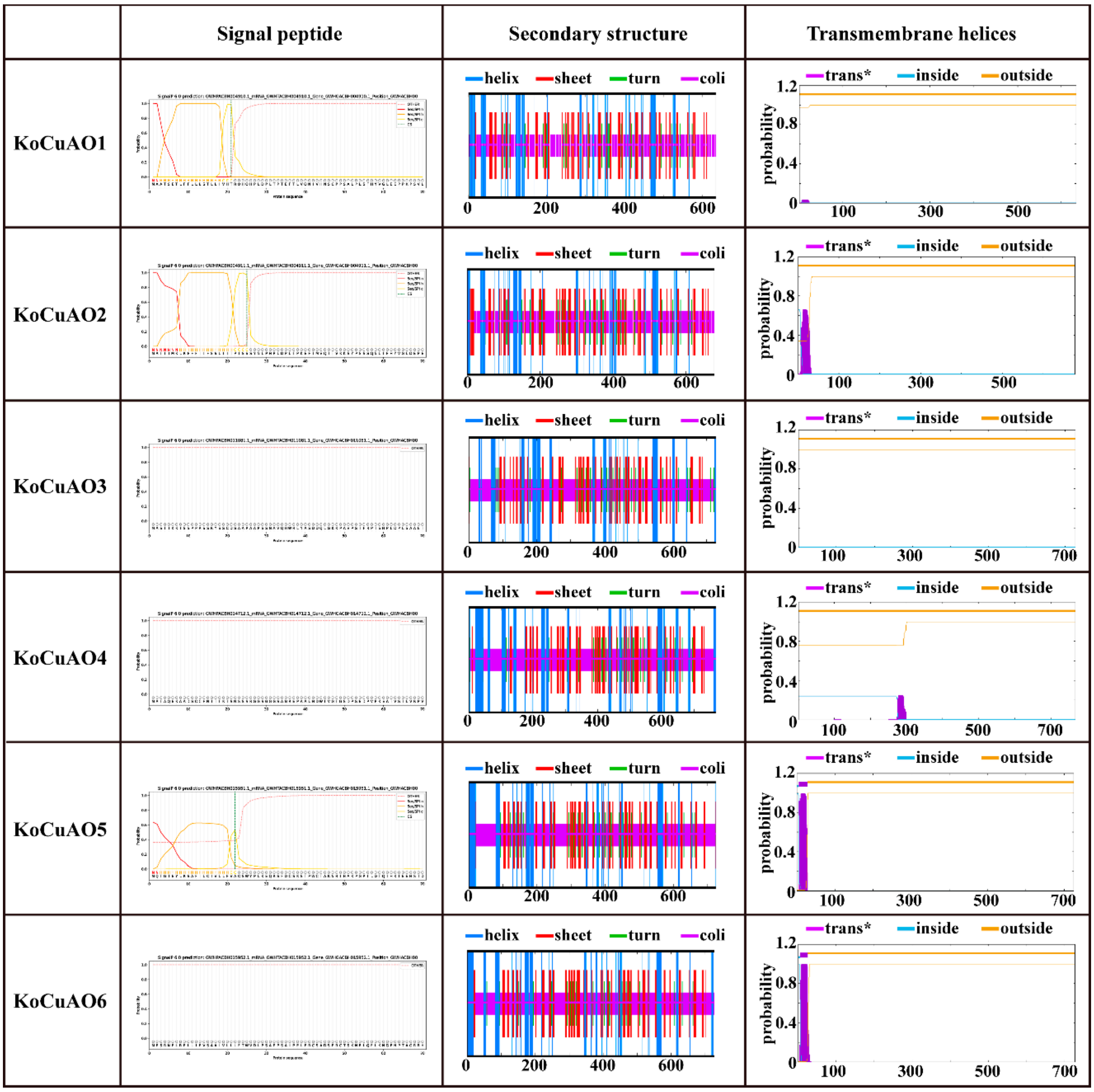


Figure S1. A full-size figure of the transmembrane structures of the KoCuAO1-6 proteins. The validity of the transmembrane structures was confirmed through the utilization of the SOPMA/prabi programme. Trans* indicated the transmembrane.
